# Protective Effects of *Alisma orientale* Extract against Hepatic Steatosis via Inhibition of Endoplasmic Reticulum Stress

**DOI:** 10.3390/ijms161125944

**Published:** 2015-11-02

**Authors:** Min-Kyung Jang, Yu-Ran Han, Jeong Soo Nam, Chang Woo Han, Byung Joo Kim, Han-Sol Jeong, Ki-Tae Ha, Myeong Ho Jung

**Affiliations:** 1 Division of Longevity and Biofunctional Medicine, School of Korean Medicine, Pusan National University, Yangsan-si 626-870, Korea; ckone0506@hanmail.net (M.-K.J.); coolyuran@hotmail.com (Y.-R.H.); hidejsugi@naver.com (J.S.N.); vision@pusan.ac.kr (B.J.K.); 2 Healthy Aging Korean Medical Research Center, School of Korean Medicine, Pusan National University, Yangsan-si 626-870, Korea; yeast10@hanmail.net (C.W.H.); jhsol33@pusan.ac.kr (H.-S.J.); hagis@pusan.ac.kr (G.-T.H.)

**Keywords:** *Alisma orientale*, endoplasmic reticulum stress, hepatic steatosis, triglyceride accumulation, lipogenesis, very low-density lipoprotein receptor, ApoB secretion

## Abstract

Endoplasmic reticulum (ER) stress is associated with the pathogenesis of hepatic steatosis. *Alisma orientale* Juzepzuk is a traditional medicinal herb for diuretics, diabetes, hepatitis, and inflammation. In this study, we investigated the protective effects of methanol extract of the tuber of *Alisma orientale* (MEAO) against ER stress-induced hepatic steatosis *in vitro* and *in vivo*. MEAO inhibited the tunicamycin-induced increase in luciferase activity of ER stress-reporter constructs containing ER stress response element and ATF6 response element. MEAO significantly inhibited tunicamycin-induced ER stress marker expression including GRP78, CHOP, and XBP-1 in tunicamycin-treated Human hepatocellular carcinoma (HepG2) cells and the livers of tunicamycin-injected mice. It also inhibited tunicamycin-induced accumulation of cellular triglyceride. Similar observations were made under physiological ER stress conditions such as in palmitate (PA)-treated HepG2 cells and the livers of high-fat diet (HFD)-induced obese mice. MEAO repressed hepatic lipogenic gene expression in PA-treated HepG2 cells and the livers of HFD obese mice. Furthermore, MEAO repressed very low-density lipoprotein receptor (VLDLR) expression and improved ApoB secretion in the livers of tunicamycin-injected mice or HFD obese mice as well as in tunicamycin or PA-treated HepG2 cells. Alismol, a guaiane-type sesquiterpenes in *Alisma orientale*, inhibited GRP78 expression in tunicamycin-treated HepG2 cells. In conclusion, MEAO attenuates ER stress and prevents hepatic steatosis pathogenesis via inhibition of expression of the hepatic lipogenic genes and VLDLR, and enhancement of ApoB secretion.

## 1. Introduction

Nonalcoholic fatty liver disease (NAFLD) is characterized by excessive lipid accumulation in the liver without chronic alcohol consumption. It encompasses diverse hepatic disorders ranging from hepatic steatosis to nonalcoholic steatohepatitis (NASH), fibrosis, and cirrhosis. The prevalence of NAFLD has been increasing worldwide and coincides with the frequency of metabolic disorders including obesity, type 2 diabetes, and hyperlipidemia. Hepatic steatosis refers to over-accumulation of triglyceride (TG) in hepatocytes. TG accumulation results from increased lipid delivery to the liver, increased *de novo* lipogenesis, and reduced fatty acid (FA) oxidation, reduced lipolysis, and decreased lipid secretion [1,2]. According to “two-hit” hypothesis, the progression of NAFLD occurs in two steps. The first step is the accumulation of TG in the liver, and the second step involves additional pathophysiological insults, including oxidative stress, inflammation, and cell death [1,2].

*Alisma orientale* Juzepzuk is widely cultivated in China, Japan, and Korea. It has been used in clinical practice for the treatment of edema, dysuria, and diarrhea, and has also been prescribed as an important component of herbal combinational therapy for the treatment of hypertension, hyperlipidemia, and hyperglycemia. Furthermore, many studies have reported that it has therapeutic potential for inflammation [3], allergy [4], and oxidative stress [5]. A recent study has shown that *Alisma orientale* has a protective effect against nonalcoholic fatty liver induced by high fat diet (HFD) [6]. However, the mechanism involved in this protective effect has not been characterized. The chemical constituents of *Alisma orientale* include sesquiterpenes, protostane-type triterpenes, and guaiane-type and kaurane-type diterpenes [7].

The endoplasmic recticulum (ER) is an intracellular organelle that regulates lipid production, protein synthesis for most cellular organelles, and Ca^2+^ storage [8,9]. Different stimuli that disrupt ER homeostasis increases the accumulation of unfolded proteins in the ER, which leads to ER stress. To solve ER stress, unfolded protein response (UPR) is activated. The UPR attenuates protein translation, degrades unfolded proteins, and increases protein folding capacity of the ER [10]. Chronic or increased ER stress leads to the pathogenesis of multiple diseases including diabetes [11]. Recently, it was reported that ER stress is associated with the development of hepatic steatosis [12]. ER stress disturbs hepatic lipid metabolism by regulating lipogenic gene expression and apolipoprotein secretion, and by promoting insulin resistance. Furthermore, ER stress activates Nrf2, JNK, and NFκ-B pathways, which play important roles in inflammatory process [13].

Although *Alisma orientale* was found to protect against HFD-induced hepatic steatosis in rat [6], the underlying mechanism was not characterized. Furthermore, it remains unclear whether *Alisma orientale* extract can attenuate ER stress, a major contributor of hepatic steatosis. Therefore, this study was designed to investigate the protective effect of *Alisma orientale* against ER stress and hepatic steatosis *in vitro* and *in vivo*. To this end, the experiments were conducted in Human hepatocellular carcinoma (HepG2) cells and mice treated with tunicamycin (pharmacologic ER stress inducer), palmitate-treated HepG2 cells, and HFD obese animals. Furthermore, the molecular mechanisms of protective effects against ER stress-induced hepatic steatosis were characterized.

## 2. Results

### 2.1. Methanol Extract of the Tuber of Alisma orientale (MEAO) Exerts a Protective Effect against Endoplasmic Reticulum (ER) Stress and Alleviates Intracellular Triglyceride Levels in Human Hepatocellular Carcinoma (HepG2) Cells 

**Figure 1:**
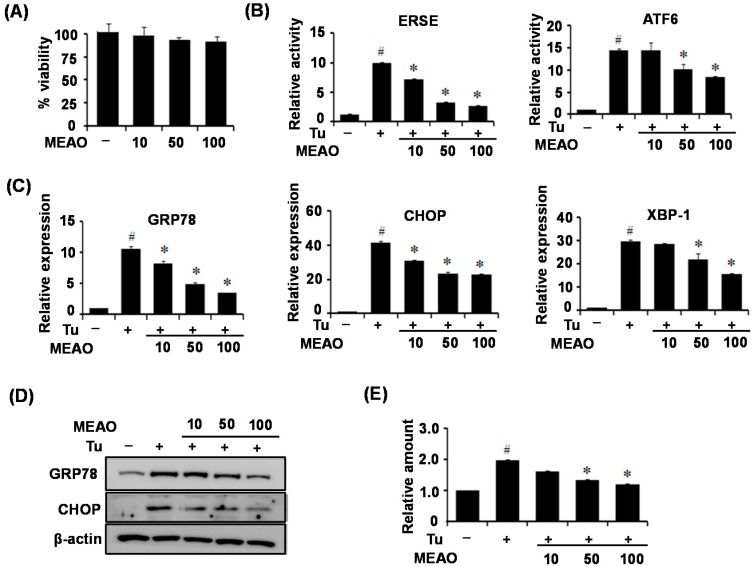
Methanol Extract of the Tuber of Alisma orientale (MEAO) protects against endoplasmic reticulum (ER) stress and alleviates intracellular triglycerides in human hepatocellular carcinoma (HepG2) cells. (**A**) MTT assay; (**B**) HepG2 cells were transfected with the ER stress response element (ERSE) reporter or activating transcription factor 6 (ATF6) reporter, and treated with tunicamycin (Tu) (2 μg/mL) in the presence of different concentrations (10, 50, and 100 μg/mL) of MEAO for 6 h. Luciferase activities were measured. Values are expressed as mean ± SEM. (*n* = 3 independent experiments). HepG2 cells were pretreated with different concentractions of MEAO for 16 h, and were treated with dimethyl sulfoxide (DMSO), or tunicamycin (Tu) (2 μg/mL) for 6 h; (**C**) Quantitative Polymerase Chain Reaction (q-PCR) analysis of GRP78, CHOP and XBP-1 mRNA splicing (*n* = 3 independent experiments); (**D**) Western blot analysis of GRP78 and CHOP. A representative image from three independent experiments is shown; (**E**) Measurement of intracellular triglycerides. GRP78, Glucose regulated protein 78; CHOP, C/EBP homolog protein; XBP-1, X-box-binding protein-1. –, without tunicamycin; +, with tunicamycin. Values are expressed as the mean ± SEM (*n* = 3 independent experiments). ^#^ *p* < 0.05 compared with untreated control. * *p* < 0.05 compared with tunicamycin-treated control.

The MTT assay indicated that MEAO was not cytotoxic to HepG2 cells within the range of 100 μg/mL (Figure 1A). To investigate the ability of MEAO to attenuate ER stress, we first examined the inhibitory effects of the extract on the luciferase activity of reporter constructs containing an ER stress response element (ERSE) and ATF6 response element in tunicamycin-treated HepG2 cells. While tunicamycin treatment increased the luciferase activity of the reporters, MEAO effectively blocked the tunicamycin-induced increase in luciferase activity (Figure 1B). Next, we determined the protective effect of MEAO against ER stress and cellular triglyceride accumulation in tunicamycin-treated HepG2 cells. Tunicamycin treatment increased the mRNA levels of ER stress markers including GRP78, CHOP and spliced XBP-1, which is a representative molecule of three UPR pathways. We observed that MEAO significantly decreased the expression of tunicamycin-induced ER stress markers (Figure 1C). Consistent with the mRNA levels, the protein levels of ER stress markers, GRP78 and CHOP also decreased after MEAO administration (Figure 1D). Next, we examined the intracellular triglyceride levels in HepG2 cells. As shown in Figure 1E, triglyceride measurements revealed that tunicamycin increased the level of intracellular triglycerides, whereas MEAO inhibited tunicamycin-induced increase in triglyceride levels. Taken together, these results indicate that MEAO exerts a protective effect against ER stress and alleviates intracellular triglyceride accumulation in HepG2 cells.

### 2.2. MEAO Prevents Hepatic ER Stress and Triglyceride Accumulation in Tunicamycin-Injected Mice

**Figure 2:**
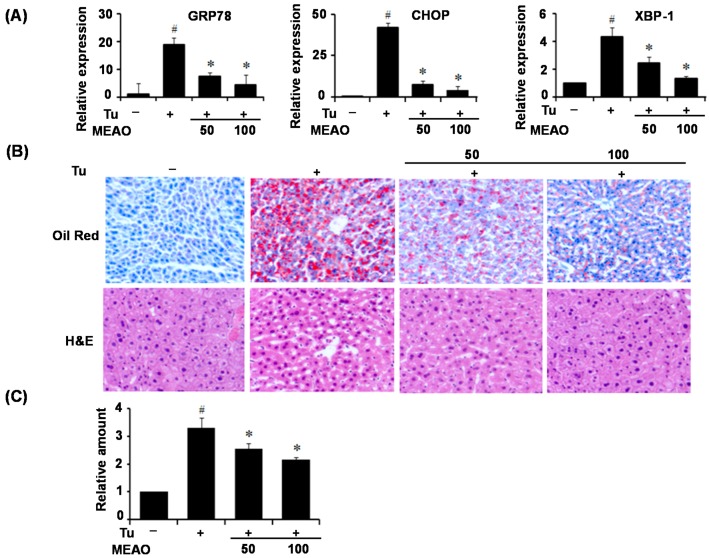
MEAO prevents hepatic endoplasmic recticulum (ER) stress and lipid accumulation in tunicamycin-injected mice. C57BL/6 mice were administrated with different doses of MEAO for 2 days, and then were injected intraperitoneally with dimethyl sulfoxide (DMSO) or tunicamycin (Tu). (**A**) q-PCR analysis of GRP78, CHOP and XBP-1 mRNA splicing (*n* = 7 mice per group); (**B**) Oil Red O staining and hematoxylin and eosin (H&E) staining of liver sections (magnification ×200); and (**C**) Measurement of hepatic triglycerides. GRP78, Glucose regulated protein 78; CHOP, C/EBP homolog protein; XBP-1, X-box-binding protein-1. –, without tunicamycin; +, with tunicamycin. Values are expressed as mean ± SEM. (*n* = 7 mice per group). ^#^ *p* < 0.05 compared with untreated control. * *p* < 0.05 compared with tunicamycin-treated control.

Next, we determined the ability of MEAO to prevent ER stress and hepatic lipid accumulation *in vivo*. C57BL/6 mice were administrated MEAO and injected with tunicamycin. As shown in Figure 2A, tunicamycin injection markedly increased the mRNA levels of ER stress markers compared to control mice, whereas MEAO significantly inhibited the tunicamycin-induced increase in ER stress markers. We then examined the effects of MEAO on hepatic steatosis in the mice. Oil red O-staining was used to assess lipid accumulation in the livers of tunicamycin-treated mice. As shown in Figure 2B, Oil-red O staining of liver sections revealed that tunicamycin injection increased hepatic triglyceride accumulation, whereas MEAO reduced the tunicamycin-induced triglyceride accumulation (Figure 2B). Consistent with this, MEAO significantly reduced tunicamycin-induced cytoplasmic lipid droplets in the liver, as revealed by hematoxylin and eosin (H&E) staining (Figure 2B). The triglyceride content was also reduced in MEAO-treated mice compared with tunicamycin control mice (Figure 2C). Taken together, these results indicate that MEAO attenuates ER stress and ameliorates ER stress-induced hepatic steatosis *in vivo*.

### 2.3. MEAO Prevents ER Stress and Intracellular Triglyceride Level Increase in Palmitate-Incubated HepG2 Cells

**Figure 3:**
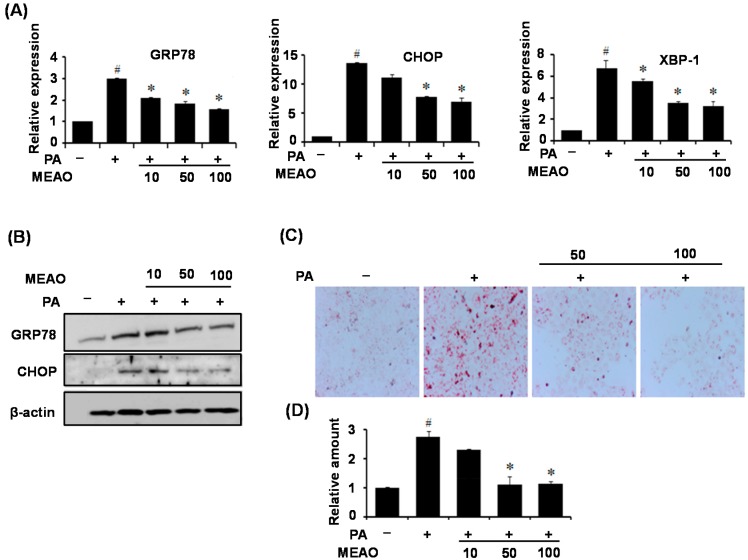
MEAO prevents palmitate (PA)-induced ER stress and alleviates intracellular triglyceride accumulation in HepG2 cells. HepG2 cells were pretreated with different concentrations of MEAO for 16 h, and were treated with dimethyl sulfoxide (DMSO), or 400 μM palmitate (PA) for 48 h. (**A**) q-PCR analysis of GRP78, CHOP, and XBP-1 mRNA splicing (*n* = 3 independent experiments); (**B**) Western blot analysis of GRP78 and CHOP. A representative image from three independent experiments is shown; (**C**) Oil Red O staining in the cells; (magnification ×200); and (**D**) Measurement of intracellular triglycerides. GRP78, Glucose regulated protein 78; CHOP, C/EBP homolog protein; XBP-1, X-box-binding protein-1. –, without palmitate; +, with palmitate. Values are expressed as mean ± SEM (*n* = 3 independent experiments). ^#^ *p* < 0.05 compared with untreated control. * *p* < 0.05 compared with palmitate-treated control.

To examine physiological relevance of MEAO, its protective effects against ER stress and cellular triglyceride accumulation were measured in HepG2 cells treated with palmitate (PA) in the presence of MEAO. As shown in Figure 3A, PA treatment increased the mRNA levels of ER stress markers in HepG2 cells, whereas MEAO significantly decreased ER stress marker induction by PA. Consistent with the mRNA levels, the protein levels of ER stress markers decreased after MEAO administration (Figure 3B). Next, we examined the levels of intracellular triglyceride in the cells. PA treatment increased intracellular triglycerides, whereas MEAO inhibited the PA-induced increase as revealed by Oil red O staining (Figure 3C) and triglyceride measurements (Figure 3D). Taken together, these results suggest that MEAO prevents PA-induced ER stress and alleviates intracellular triglyceride accumulation in HepG2 cells.

### 2.4. MEAO Extract Inhibits Hepatic ER Stress and Hepatic Steatosis in HFD Obese Mice

**Figure 4:**
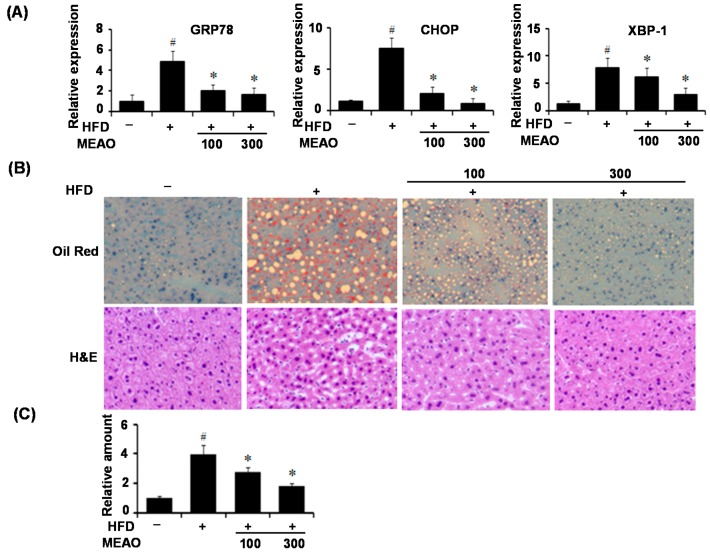
MEAO inhibits hepatic endoplasmic recticulum (ER) stress and lipid accumulation in the liver of high-fat diet (HFD) obese mice. C57BL/6J mice were fed a normal diet or HFD and orally administered different doses of MEAO daily for 16 weeks. (**A**) q-PCR analysis of GRP78, CHOP and XBP-1 mRNA splicing (*n* = 7 mice per group); (**B**) Oil Red O staining and hematoxylin and eosin (H&E) staining of the liver sections (magnification ×200); and (**C**) Measurement of hepatic triglycerides. GRP78, Glucose regulated protein 78; CHOP, C/EBP homolog protein; XBP-1, X-box-binding protein-1. –, normal diet; +, HFD. Values are expressed as mean ± SEM (*n* = 7 mice per group). ^#^ *p* < 0.05 compared with normal diet, * *p* < 0.05 compared with HFD control.

Next, we evaluated the protective effects of MEAO on high fat diet (HFD)-induced ER stress and hepatic steatosis. C57BL/6J mice were fed a normal diet or HFD and orally administered MEAO extracts daily for 16 weeks. As shown in Figure 4A, the mRNA levels of ER stress markers were markedly increased in the livers of HFD obese mice compared to lean control mice. However, MEAO significantly inhibited this increase. To examine the effect of MEAO on hepatic steatosis, we determined the degree of lipid accumulation in the livers of HFD mice by Oil red O-staining. As shown in Figure 4B, Oil-red O staining of liver sections revealed that MEAO reduced HFD-induced lipid accumulation in the mice. Consistent with this, MEAO significantly reduced HFD-induced cytoplasmic lipid droplets in the liver, as revealed by H&E staining (Figure 4B). Triglyceride measurements also showed that lipid accumulation was reduced in MEAO-treated HFD mice compared with HFD control mice (Figure 4C). Consistent with the alleviation of hepatic triglyceride, administration of MEAO reduced the blood levels of triglycerides and free fatty acids induced by HFD (data not shown). These results suggest that MEAO inhibits ER stress and ameliorates hepatic steatosis in HFD-induced obese mice.

### 2.5. MEAO Inhibits the Expression of Lipogenic Genes in PA-Incubated HepG_2_ Cells and in the Liver of HFD Obese Mice

**Figure 5:**
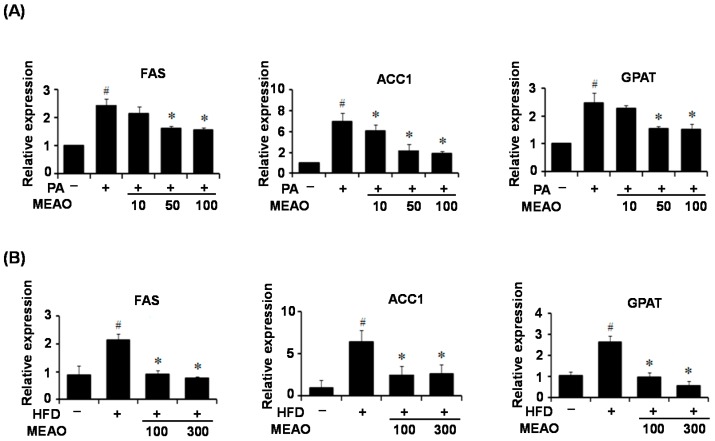
MEAO represses the expression of lipogenic genes in palmitate (PA)-treated HepG2 cells and in the livers of high-fat diet (HFD) obese mice. (**A**) q-PCR analysis of fatty acid synthase (FAS), acetyl-coenzyme A carboxylase 1 (ACC1) and glycerol-3-phosphate acyltransferase (GPAT) in palmitate-treated HepG2 cells. –, without palmitate; +, palmitate. Values are expressed as the mean ± SEM (*n* = 3 independent experiments). ^#^ *p* < 0.05 compared with untreated control. * *p* < 0.05 compared with tunicamycin-treated control; and (**B**) q-PCR analysis of FAS, ACC1 and GPAT in the livers of HFD obese mice. –, normal diet; +, HFD. Values are expressed as mean ± SEM (*n* = 7 mice per group). ^#^ *p* < 0.05 compared with normal diet. * *p* < 0.05 compared with HFD control.

It has been reported that hepatic steatosis is caused by increased lipogenesis, decreased fatty acid oxidation, decreased lipid secretion, and increased lipoprotein uptake [1,2]. ER stress upregulates the expression of major lipogenic genes, contributing to lipid accumulation in the liver [14,15]. To investigate the mechanism by which MEAO improves ER stress-induced lipid accumulation, we examined the effect of MEAO on the expression of triglyceride synthesis-associated genes in tunicamycin-incubated HepG2 cells and in the livers of tunicamycin-injected mice. The lipoogenic genes including fatty acid synthase (FAS), acetyl-coenzyme A carboxylase 1 (ACC1), and diacylglycerol acyltransferase (DGAT) decreased in tunicamycin-treated HepG2 cells and mice even though the mice had hepatic steatosis (data not shown), which was consistent with previous reports [16,17]. Therefore, we could not evaluate the anti-lipogenic effects of MEAO in tunicamycin-treated HepG2 cells and mice. We then examined the expression of lipogenic genes in PA-incubated HepG2 cells and in the livers of HFD mice. The expression of lipogenic genes including FAS, ACC1, and DGAT, increased in PA-treated HepG2 cells (Figure 5A) and in the livers of HFD obese mice (Figure 5B), whereas MEAO significantly reduced their expression. These results indicate that MEAO downregulates the expression of hepatic lipogenic genes and can inhibit lipid accumulation in the liver.

### 2.6. MEAO Represses very Low-Density Lipoprotein Receptor (VLDLR) Expression and Enhances ApoB Secretion

**Figure 6:**
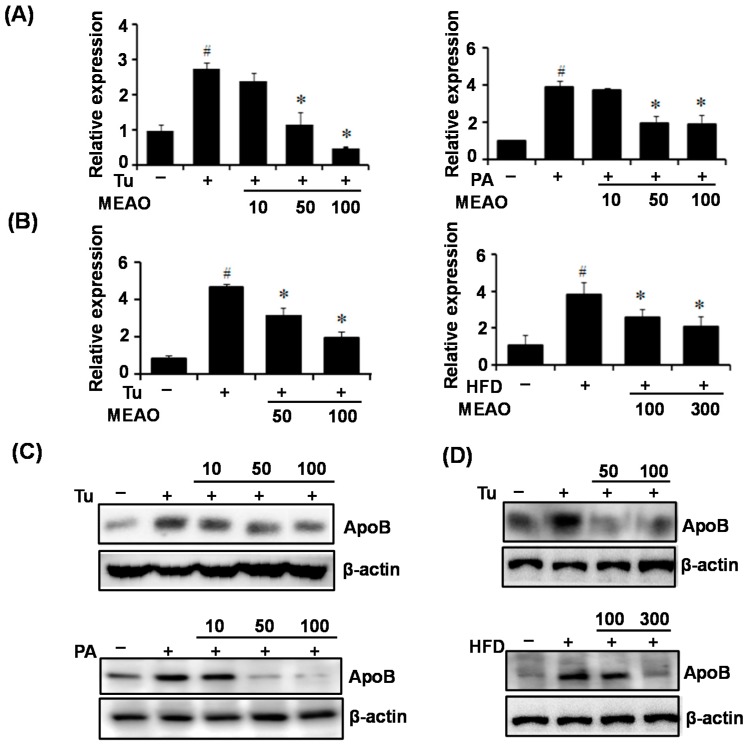
MEAO represses very low-density lipoprotein receptor (VLDLR) expression and increases apolipoprotein B (ApoB) secretion in tunicamycin (Tu) or palmitate (PA)-treated HepG2 cells, and in the livers of tunicamycin-injected or high-fat diet (HFD) obese mice. (**A**) q-PCR analysis of VLDLR in tunicamycin or palmitate-treated HepG2 cells. –, without tunicamycin or palmitate; +, with tunicamycin or palmitate. Values are expressed as the mean ± SEM (*n* = 3 independent experiments). ^#^ *p* < 0.05 compared with untreated control. * *p* < 0.05 compared with tunicamycin or palmitate-treated control; (**B**) q-PCR analysis of VLDLR in the liver of tunicamycin-injected or HFD obese mice. –, without tunicamycin; +, with tunicamycin (left). –, normal diet; +, HFD (right). Values are expressed as mean ± SEM (*n* = 7 mice per group). ^#^ *p* < 0.05 compared with untreated control or normal diet. * *p* < 0.05 compared with tunicamycin-injected or HFD control mice; (**C**) Western blot analysis of ApoB in the cell lysates of tunicamycin or palmitate-treated HepG2 cells. –, without tunicamycin or palmitate; +, with tunicamycin or palmitate. A representative image from three independent experiments is shown; (**D**) Western blot analysis of ApoB in the livers of tunicamycin-injected mice or HFD obese mice. –, without tunicamycin; +, with tunicamycin (upper). –, normal diet; +, HFD (bottom). A representative picture from 7 mice is shown.

Recently, it was reported that tunicamycin-induced ER stress increased the expression of very low-density lipoprotein receptor (VLDLR), which could contribute to hepatic steatosis [17]. Therefore, we investigated whether MEAO blocks the ER stress-induced increase in VLDLR expression in HepG2 cells and mice. As shown in Figure 6A, VLDLR expression increased in tunicamycin or palmitate incubated-HepG2 cells, whereas MEAO reduced the increase in a dose-dependent way. Similar observations were made in the liver of tunicamycin-injected mice and HFD mice (Figure 6B). Furthermore, it was demonstrated that ER stress inhibited the secretion of ApoB, a secretary protein involved in the transfer of lipids out of hepatic cells, and increased intracellular triglyceride accumulation [18]. Therefore, we investigated whether MEAO affected the inhibition of ApoB secretion in tunicamycin or PA-treated HepG2 cells. As shown in Figure 6C, treatment with tunicamycin or PA increased the intracellular ApoB levels in the cell lysates, suggesting that ApoB secretion is inhibited by treatment with tunicamycin or palmitate. However, MEAO decreased the intracellular ApoB level in the cell lysates (Figure 6C). These data indicate that MEAO reduces the inhibition of ApoB secretion induced by treatment with tunicamycin or palmitate. To further confirm this mechanism, ApoB levels were examined in the livers of tunicamycin-injected or HFD obese mice. As shown in Figure 6D, the ApoB levels increased in the livers of both tunicamycin-injected and HFD mice compared to the control mice. However, MEAO administration markedly decreased the hepatic ApoB levels. Together, these results suggest that MEAO restores the inhibition of ApoB secretion by ER stress.

### 2.7. MEAO Inhibits ER Stress-Induced Inflammation

**Figure 7:**
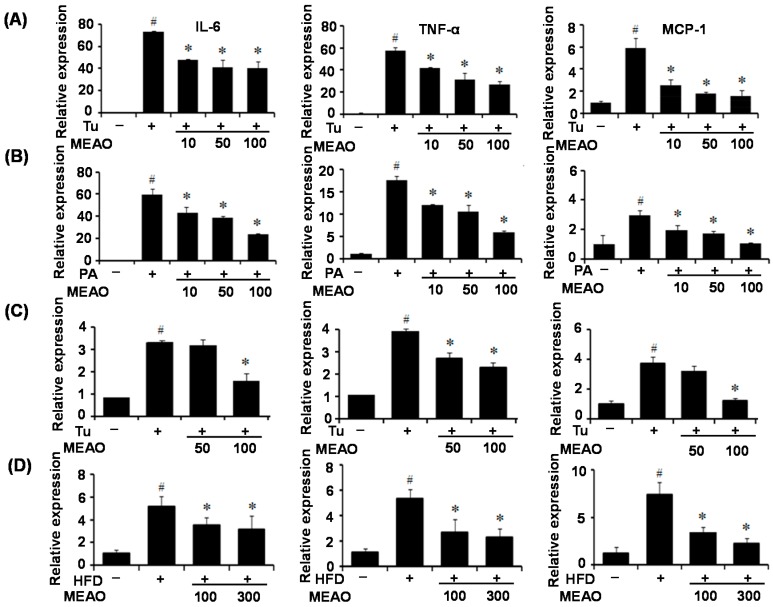
MEAO prevents endoplasmic recticulum (ER) stress-induced inflammation. (**A**,**B**) q-PCR analysis of interleukin (IL)-6, tumor necrosis factor (TNF)-α and monocyte chemotactic protein (MCP)-1 in tunicamycin (Tu) (**A**) or palmitate (PA) (**B**)-treated HepG2 cells. –, without tunicamycin or palmitate; +, with tunicamycin or palmitate. Values are expressed as the mean ± SEM (*n* = 3 independent experiments). ^#^ *p* < 0.05 compared with untreated control. * *p* < 0.05 compared with tunicamycin or palmitate-treated control. (**C**,**D**) q-PCR analysis of IL-6, TNF-α and MCP-1 in the liver of tunicamycin-injected mice (**C**) or high-fat diet (HFD) obese mice (**D**). –, without tunicamycin; +, with tunicamycin (C). –, normal diet; +, HFD (D). Values are expressed as mean ± SEM (*n* = 7 mice per group). ^#^ *p* < 0.05 compared with untreated control or normal diet. * *p* < 0.05 compared with tunicamycin-injected or HFD control mice.

It has been demonstrated that ER stress contributes to inflammation through the activation of NFκB [19]. Severe fatty liver is occasionally accompanied by inflammation, a situation referred to as steatohepatitis [1]. Therefore, we investigated whether MEAO inhibits the inflammation associated with ER stress. As shown in Figure 7, treatment with tunicamycin (Figure 7A) or PA (Figure 7B) increased the expression of inflammatory genes including interleukin (IL)-6, tumor necrosis factor (TNF)-α, and monocyte chemotactic protein (MCP)-1 in HepG2 cells, whereas MEAO prevented their increased expression. Similar results were observed in the livers of tunicamycin-injected mice (Figure 7C) and HFD mice (Figure 7D), suggesting that MEAO inhibits ER stress-induced inflammation.

### 2.8 Alismol Inhibited GRP78 Expression in Tunicamycin-Treated HepG2 Cells

**Figure 8:**
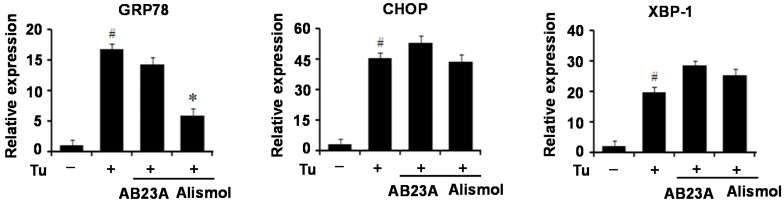
Alismol inhibited GRP78 expression in tunicamycin (Tu)-treated HepG2 cells. HepG2 cells were pretreated with alismol (100 μM) or alisol B23-acetate (10 μM) (AB23A) for 16 h, and were treated with dimethyl sulfoxide (DMSO), or tunicamycin (Tu) (2 μg/mL) for 6 h. q-PCR analysis of GRP78, CHOP and XBP-1 mRNA splicing (*n* = 3 independent experiments). GRP78, Glucose regulated protein 78; CHOP, C/EBP homolog protein; XBP-1, X-box-binding protein-1. –, without tunicamycin; +, with tunicamycin. ^#^ *p* < 0.05 compared with untreated control. * *p* < 0.05 compared with tunicamycin-treated control.

The chemical composition of *Alisma orientale* mainly includes guaiane-type sesquiterpenes and protostane-type triterpenes such as alisol derivatives [20]. Among the constituents of *Alisma orientale*, alismol is a sesquiterpenoid, and alisol B 23-acetate is a triterpenoid. To identify a component that contributes to the protective effect against ER stress, we examined ER stress marker proteins in tunicamycin-treated HepG2 cells in the presence of alismol or alisol B23-acetate. As shown in Figure 8, alismol efficiently decreased GRP78 expression; however, it did not affect the levels of CHOP and XBP-1. In contrast, alisol B23-actate did not decrease the expression of ER stress marker proteins.

## 3. Discussion

*Alisma orientale* is a well-known Chinese traditional medicine, which exhibits anti-inflammatory and anti-allergic properties [3,14]. Chronic ER stress has been reported to induce metabolic diseases including type 2 diabetes, hyperlipidemia, and obesity [11,12,13]. Recently, it was reported that ER stress causes the development of non-alcoholic fatty liver and alcoholic fatty liver by regulating lipid metabolism [12]. Therefore, a substance that could attenuate ER stress would be a therapeutic candidate for fatty liver disease. Reportedly, tauroursodeoxycholic acid and 4-phenylbutyric acid attenuate ER stress by increasing protein folding and trafficking, and decrease hepatic lipid accumulation in *ob*/*ob* mice [21,22,23]. Here, we demonstrated that MEAO prominently attenuated ER stress and prevented the development of the hepatic steatosis induced by ER stress.

*Alisma orientale* has been proved to display positive pharmacological effects against several diseases. However, to our knowledge no study has evaluated its protective effects against ER stress, which is a major pathogenic factor for several diseases including hepatic steatosis. Therefore, in this study, we investigated the protective effects of MEAO against ER stress, and determined whether MEAO could ameliorate ER stress-induced hepatic steatosis. We first determined the inhibitory activity of MEAO on ER stress reporters including ER stress response element or ATF6 response element. MEAO efficiently inhibited the tunicamycin-induced increase in luciferase activity of the reporters. Then, the protective effects against ER stress and ER stress-induced hepatic steatosis were examined *in vitro and in vivo*. For the *in vitro* study, HepG2 cells were treated with tunicamycin in the presence of MEAO, and ER stress markers and cellular triglyceride levels were measured in the cell extracts. MEAO greatly attenuated the tunicamycin-induced increases in both ER stress marker and cellular triglyceride levels. Similarly, for the *in vivo study*, C57BL6J mice were administered MEAO for 2 days, and injected with tunicamycin for 24 h. Tunicamycin treatment increased the levels of ER stress marker proteins and triglyceride accumulation in the livers, both of which decreased in MEAO-administered mice. Taken together, the *in vitro* and *in vivo* study results indicate that MEAO attenuated ER stress and improved ER stress-induced hepatic steatosis.

We then confirmed the protective effects of MEAO in fatty acid-treated HepG2 cells and HFD obese mice. PA treatment induced ER stress in HepG2 cells as revealed by increase in ER stress markers, and increased cellular triglyceride levels. HFD also induced hepatic ER stress and increased hepatic lipid accumulation; however, MEAO treatment efficiently blocked the increase in ER stress markers and lipid accumulation. These results demonstrate that MEAO ameliorated hepatic steatosis through the inhibition of ER stress.

The ER elicits an elaborative adaptive response under pathological and/or stressful conditions, collectively known as the UPR [8,9]. The three branches of the UPR regulate the transcription of various genes involved in the expansion of ER, decrease of protein synthesis, increase of protein folding capability and degradation of terminally unfolded and misfolded proteins within the ER. Previous study reported that IRE1α contributed to the development of hepatic steatosis through upregulation of gene expression involved in fatty acid and triglyceride synthesis by XBP-1 [14]. PERK also increased the expression of the lipogenic enzymes such as FAS, ATP citrate lyase, and stearoyl-CoA desaturase-1 in the mouse mammary gland [15]. Furthermore, CHOP deficiency in mice inhibited cholestasis induced liver fibrosis [24]. According to our current results, MEAO blocked the increase in GRP78, CHOP and XBP-1x splicing respectively, suggesting that MEAO potentially acts on hepatic steatosis via inhibition of the three branches of UPR.

To investigate the mechanism involved in ER stress-mediated inhibition of lipid accumulation, we first examined the expression of lipogenic genes in the livers of tunicamycin-injected mice because ER stress regulates lipid metabolism-genes. Consistent with previous reports [16,17], tunicamycin treatment decreased the expression of lipogenic genes including FAS, ACC1, and GAPT despite the presence of hepatic steatosis. However, this was not significantly affected by MEAO. These results suggest that the rescue of tunicamycin-induced hepatic steatosis by MEAO was not due to the inhibition of lipogenesis. However, the increased expression of lipogenic genes in PA-treated HepG2 cells and in the liver of HFD obese mice was inhibited by MEAO, suggesting that MEAO potentially ameliorates fatty acid-induced hepatic steatosis through inhibition of lipogenesis.

Furthermore, it was known that VLDLR mediates APOE-containg VLDL uptake into hepatic cells, and increased intracellular triglyceride accumulation. ApoB is a secretary protein involved in the transfer of lipids out of hepatic cells. ER stress has been shown to increase VLDLR expression and inhibit ApoB secretion, which might be a primary mechanism of tunicamycin-induced hepatic steatosis under conditions of suppressed lipogenesis [17,18]. Therefore, we investigated VLDLR expression and ApoB secretion in tunicamycin-treated HepG2 cells and mice. Tunicamycin treatment increased VLDL expression and intracellular ApoB protein levels in the livers of mice as well as in HepG2 cells. However, the increases in VLDL expression and intracellular ApoB levels were inhibited by MEAO. The suppressive effects of MEAO on VLDL expression and intracellular ApoB levels were also observed in PA-incubated HepG2 cells and in the livers of HFD obese mice, indicating that MEAO suppresses VLDLR expression, decreases uptake of APOE-containing VLDL, and also inhibits ApoB secretion, which might cause the attenuation of triglyceride accumulation. Together, these results suggest that MEAO attenuated ER stress and ameliorated hepatic steatosis via the downregulation of VLDLR expression, and the improvement of ApoB secretion under conditions of suppressed lipogenesis.

Inflammation has been demonstrated to be an important mechanism of hepatic steatosis and to exacerbate liver injury and NASH [1,2]. In addition, it has been reported that ER stress causes inflammation [13]. Therefore, we investigated whether MEAO alleviates hepatic ER stress-associated inflammation *in vitro* and *in vivo*. Pro-inflammatory cytokines, such as IL-6, TNF-α, and MCP1 were induced by treatment with tunicamycin and PA. However, MEAO prevented the increase of inflammatory cytokine expression. These results indicate that MEAO ameliorated the ER stress induced-inflammation associated with hepatic steatosis.

Terpenoid is a main chemical component of *Alisma orientale* along with protostane-type triterpenes, guaiane-type sesquiterpenes, and kauranetype diterpenes [20]. Many terpenes has been isolated from *Alisma orientale* [25,26] which were known to have various functions including anti-type I–IV allergic response activity, anti-inflammatory activity, anti-hepatitis B virus activity, anti-tumor activity, and anti-complementarity activity [3,4,5]. Among these components, alismol is a guaiane-type sesquiterpenes that has been shown to inhibit nitric oxide synthesis in RAW 264.7 cells stimulated with interferon-gamma plus lipopolysaccharide and to suppress mainly Ca^2+^ influx through a voltage-dependent Ca^2+^ channel [27,28]. In this study, alismol significantly blocked the increase in GRP78 expression mediated by tunicamycin, whereas it did not affect the levels of CHOP and XBP-1c, suggesting that alismol might represent one of the active components of MEAO responsible for anti-ER stress and hepatic steatosis which might exert a protective effect against ER stress through the inhibition of ATF6-GRP78 pathway among the three branches of UPR.

In conclusion, MEAO efficiently attenuated ER stress and prevented the development of hepatic steatosis through inhibition of the expression of hepatic lipogenic genes and of VLDLR, and enhancement of ApoB secretion. Our experimental evidence supports the use of MEAO as a potential therapeutic agent for the treatment of ER stress-induced diseases.

## 4. Experimental Section

### 4.1. Reagents

Tunicamycin and palmitate were purchased from Sigma-Aldrich (St. Louis, MO, USA). Alismol and alisol B23-acetate were purchased from Core Science (Seoul, Korea). Antibodies against GRP78 and CHOP were purchased from Santa Cruz Biotechnology (Santa Cruz, CA, USA). Antibody against ApoB 100 was purchased from Cell Signaling Technology (Beverly, MA, USA).

### 4.2. Cell Lines

Human hepatocellular carcinoma cells (HepG2) were purchased from the American Type Culture Collection (Manassas, VA, USA). HepG2 cells were cultured in Dulbecco’s modified Eagle’s medium (DMEM) containing glucose (Invitrogen, Carlsbad, CA, USA) supplemented with 10% (*v*/*v*) fetal bovine serum (Invitrogen).

### 4.3. Preparation of Alisma Orientale Extract

Dried tubers of *Alisma orientale* Juzepzuk used in this study was purchased from Naemomea Dah Corporation (Ulsan, Korea, in July 2014), and was identified by Professor Ki-Tae Ha, School of Korean Medicine, Pusan National University (Korea). The whole plant voucher specimen (number: pnuhc001) was registered and deposited at the School of Korean Medicine, Pusan National University. Dried specimen (400 g) was ground into power, and extracted three times with methanol (1 L × 3) for 3 h at room temperature, and filtered through a filter paper. The filtrate was evaporated *in vacuo* at 40 °C to produce a methanol extract of *Alisma orientale* (MEAO, 58.76 g). Appropriate amount of the extract was dissolved in dimethyl sulfoxide (DMSO) prior to experiment.

### 4.4. Cell Viability Assay

Cell viability was measured by a quantitative colorimetric assay with 3-(4,5-dimethylthiazol-2-yl)-2,5-diphenyltetrazoli-um bromide (MTT). HepG2 cells were plated in 96-well plates at a density of 5000 cells/well and then cultured for 24 h. After incubation the cells with various concentrations of MEAO (10, 50, and 100 µg/mL) for 16 h, MTT solution (2.0 mg/mL) was added to each well for 4 h. DMAO was added to each well after removing the media, and the optical density (OD) was measured at 540 nm with a microplate reader.

### 4.5. Reporter Assay

To determine the protective activity against ER stress, HepG2 cells (2 × 10^4^ cells/well in a 96-well plate) were transfected with an ERSE-Luc reporter or ATF6-Luc reporter using a Cignal™ Reporter Assay kit (Qiagen, Valencia, CA, USA). The ERSE-Luc and ATF6-Luc were purchased from Addgene (Cambridge, MA, USA). The consensus ERSE is 5′-CCAAT(N9)CCACG and ATF6 sequence is 5′-TCGAGACAGGTGCTGACGTGGCGATTCC. The cells were then incubated with tunicamycin (2 μg/mL) in the presence of the MEAO and then the luciferase activities were determined with a Dual-Glo Luciferase assay system kit (Promega, Madison, WI, USA).

### 4.6. Animal Experiments

C57BL/6 mice were purchased from Central Lab. Animal Inc. (Seoul, Korea). The animals were maintained on a 12 h light/dark cycle under 21–23 °C of temperature and 40%–60% of humidity. For the tunicamycin-induced ER stress animal model, mice at 8 weeks of age were randomly divided into four groups (*n* = 7) with equal mean body weights. The mice were administrated MEAO (50 mg/kg body weight and 100 mg/kg body weight) for 2 days and then injected intraperitoneally with DMSO or tunicamycin at a dose of 1 mg/kg body weight for 24 h. Then, the mice were administrated with MEAO for additional 2 days and sacrificed. For high fat diet (HFD) obese mice, the mice at 6 weeks of age were randomly divided into four groups (*n* = 7) with equal mean body weights: lean control (distilled water-treated) group, high-fat diet (distilled water-treated) group, high-fat diet and MEAO (100 mg/kg/day of body weight) group, high-fat diet and MEAO (300 mg/kg/day of body weight) group. The experimental diets were the AIN93G-based on the High-fat diet containing 60% kcal fat (37.1% of saturated fat) and the control diet containing 10% kcal fat. MEAO was administered orally daily for 16 weeks. All animal experiments were approved by the Pusan National University Animal Experiment Ethics Committee and were conducted according to the institutional guidelines for the care and use of laboratory animals.

### 4.7. Histological Analysis

Liver specimens were fixed in 10% formalin and embedded in paraffin, and cutted into 3 µm sections. The liver slides were washed with 60% isopropanol for 5 min and stained with Oil-red O working solution (1.5 mg/mL Oil-red O/60% isopropanol) for 15 min at room temperature. The slides were washed with distilled water and photographed under a light microscope. For hematoxylin and eosin (H&E) staining, the liver slides were stained with hematoxylin and eosin, and the histopathology was observed under a microscope.

### 4.8. Measurement of Triglyceride Level

Liver tissues and HepG2 cells were homogenized in chloroform-methanol solution (2:1, *v*/*v*) and choloroform-methanol-H_2_O solution (8:4:3, *v*/*v*), respectively. The homogenates were incubated at room temperature for 1 h and centrifuged at 800× *g* for 10 min. The obtained bottom layer (organic phage) was dried overnight. After dissolving the dried bottom layer in ethanol, triglyceride concentrations were determined using an enzyme reaction kit (Asan pharmaceutical, Seoul, Korea) and normalized to the protein concentration.

### 4.9. Quantitative Polymerase Chain Reaction (q-PCR)

RNA was isolated from the livers of the mice and HepG2 cells with Trizol™ (Invitrogen). The cDNA was generated from 1 μg of total RNA using GoScript™ Reverse Transcription System (Promega, Madison, WI, USA) following the manufacturer’s protocol. The primers used in this study are listed (Table S1).

### 4.10. Western Blot

Equal amounts of protein (40 μg/lane) from liver homogenates and HepG2 cell lysates were resolved by 8% SDS polyacrylamide gel electrophoresis (SDS-PAGE) and transferred to polyvinylidene difluroide membranes (Millipore, Massachusetts, MA, USA). The levels of GRP78, CHOP, and actin were detected using an enhanced chemiluminescence western blot detection kit (Amersham, Uppsala, Sweden).

### 4.11. Statistical Analysis

All data are presented as mean ± SEM. Statistical comparisons were analyzed using Student’ *t*-test and one-way analysis of variance (ANOVA), followed by Tukey’s test. Values were considered statistically significant at *p* < 0.05.

## Supplementary Materials

Supplementary materials can be found at http://www.mdpi.com/1422-0067/16/11/25944/s1.

## Abbreviations

ACC1, Acetyl-CoA carboxylase1; CHOP, C/EBP homolog protein; ER stress, Endoplasmic reticulum stress; FAS, Fatty acid synthase; GRP78, Glucose regulated protein 78; HFD, High fat diet; IRE1α, Inositol-requiring kinase 1α; MEAO, Methanol extract of *Alisma orientale*; NAFLD, Nonalcoholic fatty liver disease; NASH, nonalcoholic steatohepatitis; p-eIF2α, Phosphor-eukaryotic translational initiation factor α; qPCR, Quantitative polymerase chain reaction; TNF-α, Tumor necrosis factor-α; Tu, Tunicamycin; VLDLR, very low-density lipoprotein receptor; XBP-1, X-box-binding protein-1.
