# Supplementary material for: Protective Effects of Alisma orientale Extract against Hepatic Steatosis via Inhibition of Endoplasmic Reticulum Stress
Source: Int J Mol Sci. 2015 Nov 2;16(11):26151–65. doi: 10.3390/ijms161125944 (PMC4661803; doi:10.3390/ijms161125944)
Supplement: Supplementary file 1 [file ijms-16-25944-s001.pdf]

International Journal of
*Molecular Sciences*

MDPI

# Supplementary Information

**Table S1.** List of primers for q-PCR.

| Gene | Forward Primer | Reverse Primer |
|---|---|---|
| **Human** | | |
| **Gene** | **Forward Primer** | **Reverse Primer** |
| hFAS | TCGTGGGCTACAGCATGGT | GCCCTCTGAAGTCGAAGAAGAA |
| hACC1 | CTGTAGAAACCCGGACAGTAGAAC | GGTCAGCATACATCTCCATGTG |
| hGPAT | AACCCCAGTATCCCGTCTTT | CAGTCACATTGGTGGCAAAC |
| hGRP78 | ATGATGCTGAGAAGTTTGCTGA | GGAAAGTTTACCTCCCAGCTTT |
| hCHOP | AGGGAGAACCAG GAAACG GAA ACA | TCC TGC TTG AGC CGT TCA TTC TCT |
| hXBP-1 | TGCTGAGTCCGCAGCAGGTG | GCTGGCAGGCTCTGGGGAAG |
| hTNF-$\alpha$ | TGCTTGTTCCTCAGCCTCTT | ATGGGCTACAGGCTTGTCACT |
| hIL-6 | ACTCACCTCTTCAGAACGAATTG | CCATCTTTGGAAGGTTCAGGTTG |
| hMCP-1 | CCCCAGTCACCTGCTGTTAT | TGGAATCCTGAACCCACTTC |
| hVLDLR | CTGTGTAAAGAAGACGTGTGCT | TGATTTCATGTATGCGGCATGT |
| hm18s | CGGCTACCACATCCAAGGAA | GCTGGAATTACCGCGGCT |
| **Mouse** | | |
| **Gene** | **Forward Primer** | **Reverse Primer** |
| mFAS | AGG TGG TGA TAG CCG GTA TGT | TGG GTA ATC CAT AGA GCC CAG |
| mACC1 | TGACAGACTGATCGCAGAGAAAG | TGGAGAGCCCCACACACA |
| mGPAT | CAACACCATCCCCGACATC | GTGACCTTCGATTATGCGATCA |
| mGRP78 | GAAAGGATGGTTAATGATGCTGAG | GTCTTCAATGTCCGCATCCTG |
| mCHOP | CAGTCATGGCAGCTGAGTCC | TAGGTGCCCCCAATTTCATC |
| mXBP-1 | GAG TCC GCA GCA GGT G | GTG TCA GAG TCC ATG GGA |
| mVLDLR | AGACCAATCAGACGAGTCTCTT | CTGCCGTCCTTGCAGTCAG |
| mTNF-$\alpha$ | CCCTCACACTCAGATCATCTTCT | GCTACGACGTGGGCTACAG |
| mIL-6 | TAGTCCTTCCTACCCCAATTTCC | TTGGTCCTTAGCCACTCCTTC |
| mMCP-1 | GCATCCACGTGTTGGCTCA | CTCCAGCCTACTCATTGGGATCA |
| hm18s | CGGCTACCACATCCAAGGAA | GCTGGAATTACCGCGGCT |
